# Functional and Structural Leaf Plasticity Determine Photosynthetic Performances during Drought Stress and Recovery in Two *Platanus orientalis* Populations from Contrasting Habitats

**DOI:** 10.3390/ijms21113912

**Published:** 2020-05-30

**Authors:** Violeta Velikova, Carmen Arena, Luigi Gennaro Izzo, Tsonko Tsonev, Dimitrina Koleva, Massimiliano Tattini, Olympia Roeva, Anna De Maio, Francesco Loreto

**Affiliations:** 1 Institute of Plant Physiology and Genetics, Bulgarian Academy of Sciences, Acad. G. Bonchev Str. bl. 21, Sofia 1113, Bulgaria; 2 Department of Biology, University of Naples Federico II, Via Cinthia, 80126 Naples, Italy; c.arena@unina.it (C.A.); andemaio@unina.it (A.D.M.); 3 Department of Agricultural Sciences, University of Naples Federico II, Via Università 100, 80055 Portici, Italy; luigigennaro.izzo@unina.it; 4 Institute of Biophysics and Biomedical Engineering, Bulgarian Academy of Sciences, Acad. G. Bonchev Str., bl. 21, Sofia 1113, Bulgaria; ttsonev@bio21.bas.bg (T.T.); olympia@biomed.bas.bg (O.R.); 5 Faculty of Biology, Sofia University, 1113 Sofia, Bulgaria; koleva@biofac.uni-sofia.bg; 6 Institute for Sustainable Plant Protection, Department of Biology, Agriculture and Food Sciences, The National Research Council of Italy (CNR), I-50019 Sesto Fiorentino (Florence), Italy; massimiliano.tattini@ipsp.cnr.it; 7 Department of Biology, Agriculture and Food Sciences, The National Research Council of Italy (CNR), 00185 Rome, Italy

**Keywords:** climate change, phenotypic plasticity, drought, photosynthesis, leaf structure

## Abstract

In the context of climatic change, more severe and long-lasting droughts will modify the fitness of plants, with potentially worse consequences on the relict trees. We have investigated the leaf phenotypic (anatomical, physiological and biochemical) plasticity in well-watered, drought-stressed and re-watered plants of two populations of *Platanus orientalis*, an endangered species in the west of the Mediterranean area. The two populations originated in contrasting climate (drier and warmer, Italy (IT) population; more humid and colder, Bulgaria (BG) population). The IT control plants had thicker leaves, enabling them to maintain higher leaf water content in the dry environment, and more spongy parenchyma, which could improve water conductivity of these plants and may result in easier CO_2_ diffusion than in BG plants. Control BG plants were also characterized by higher photorespiration and leaf antioxidants compared to IT plants. BG plants responded to drought with greater leaf thickness shrinkage. Drought also caused substantial reduction in photosynthetic parameters of both IT and BG plants. After re-watering, photosynthesis did not fully recover in either of the two populations. However, IT leaves became thicker, while photorespiration in BG plants further increased, perhaps indicating sustained activation of defensive mechanisms. Overall, our hypothesis, that plants with a fragmented habitat (i.e., the IT population) lose phenotypic plasticity but acquire traits allowing better resistance to the climate where they became adapted, remains confirmed.

## 1. Introduction

Physiology and life history of plants can be significantly altered by environmental factors. Increased frequency of extreme weather events is becoming an important feature of predicted climate changes, especially in the Mediterranean area [1]. The rise of CO_2_ in the atmosphere proceeds at an unfortunately rapid speed, and the associated global warming can lead to drastic changes in precipitation and reduction of available freshwater [1]. It is expected that drought in warm periods will become the most frequent climate extreme, negatively affecting terrestrial ecosystems [2]. However, the impact of drought stress on plants remains largely undetermined [3]. Plants, due to their sessile nature, have developed strategies to respond effectively to environmental changes, and to continually adapt to their local environment, thereby exploring highly diverse habitats [4]. 

Development of a wide range of plant phenotypes depending on environmental drivers is a phenomenon long known as phenotypic plasticity [5,6]. High phenotypic plasticity is associated with wide geographical distribution of a species. It may act as a barrier against climate change on both short- and long-term basis [6], and might be a trait of important adaptive significance [7,8]. 

Understanding the relative contribution of individual components of phenotypic plasticity, such as morpho-anatomical and physiological features, may help to predict plant performance/fitness under future environmental conditions [9] and the impact of global climate change on species composition and distribution [4,8,10]. 

To quantitatively assess phenotypic plasticity, different indices have been employed. Specific leaf area (SLA, the ratio between leaf area and leaf dry mass) is a main morphological index, which responds to a wide range of environmental stimuli, including drought, heat, and high light [11,12]. SLA components, such as whole-leaf thickness, thickness of adaxial/abaxial epidermis, proportion of palisade to spongy parenchyma, and mesophyll compactness [13,14], play a key role in photosynthesis, since they determine flux and distribution of photons inside leaves [15,16], and mesophyll limitation to CO_2_ diffusion to the carboxylation sites in the chloroplasts, especially under stressful conditions [17,18,19,20,21]. Mesophyll conductance (g_m_) variation may explain large inter- and intra-specific variations in photosynthetic capacity under both optimal and stressful conditions [22,23,24], especially when plants are concomitantly challenged by drought, high light and elevated temperatures [23,25,26].

Quantitative understanding of the physiological responses at the species level is fundamental to predict how vegetation will respond to future climate changes, and appraisal of photosynthetic reactions usefully characterizes plant sensitivity to stress [27,28] and onset of damage associated to reactive oxygen species (ROS) production [29]. 

Plane (*Platanus orientalis*) is a tree species almost extinct in Western Europe ecosystems [30]. Because of its hydrophilic habitat, *P. orientalis* may also be strongly affected by increasing water limitations in Eastern Europe, where it continues to thrive in natural and domesticated stands. Studying populations of planes that live in areas with different climate may help understand whether and how environmental conditions modify the phenotypic plasticity of this plant. Here, we used two populations of *P. orientalis* living in climatically different habitats in Italy and Bulgaria. In Bulgaria, *P. orientalis* populations are adapted to more humid environment, while in Italy relic populations of *P. orientalis* grow in fragmented habitats characterized by long dry summers. In a previous research we showed that under well-watered (control) conditions these populations have different isoprene emission, stomatal behavior, photosynthetic use of the absorbed light, D1 protein amount in the photosystem II (PSII) and thermostability of the thylakoid membranes [31]. We now use the same plant material to further explore the potential relationship between anatomical, physiological and biochemical plasticity [32,33] under drought stress conditions. It is postulated that species from harsher habitats are less plastic than those growing in favorable environments [34,35]. Based on this assumption, we hypothesized that the plasticity of the Italian (IT) population will be lower compared to that of the Bulgarian (BG) population. Exploring the plasticity for a wide range of anatomical, physiological and biochemical traits could contribute clarifying the mechanisms involved in the survival at the extreme environments. Improved understanding of *P. orientalis* phenotypic plasticity could assist in developing landscaping guidelines for reforestation of areas under different climatic conditions, and in preserving endangered habitats. 

## 2. Results

### 2.1. Variations in Leaf Anatomical Traits

**Figure 1:**
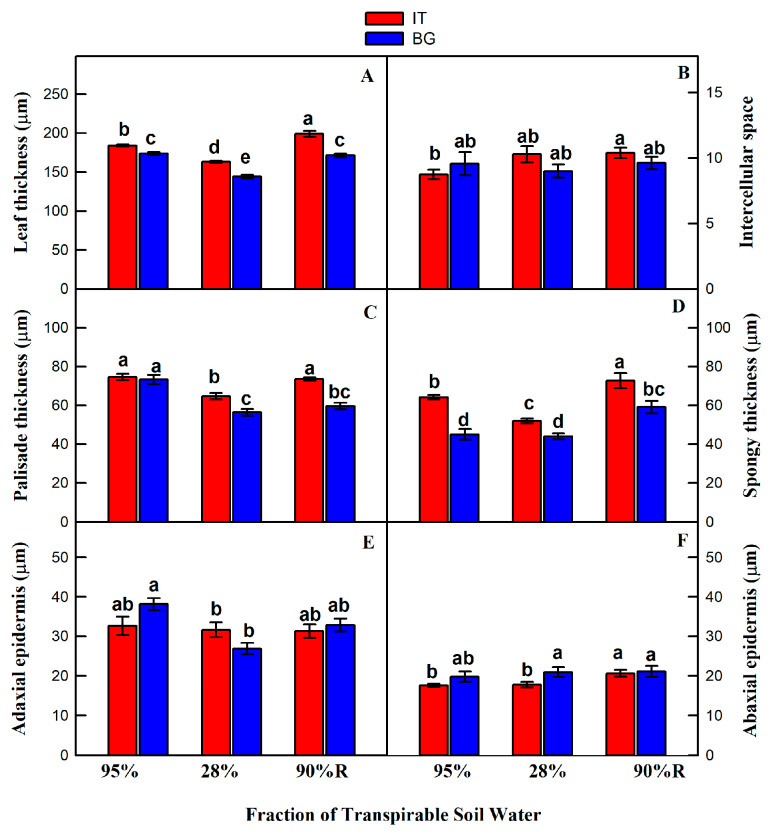
Leaf structural traits in Italian (IT, red bars) and Bulgarian (BG, blue bars) populations of *Platanus orientalis* plants under control (fraction of transpirable soil water (FTSW) = 95%) and drought (FTSW = 28%) conditions and after re-watering (FTSW = 90%R). (**A**) Leaf thickness; (**B**) relative area of mesophyll occupied by intercellular space; (**C**) palisade thickness; (**D**) spongy thickness; (**E**) adaxial epidermis; (**F**) abaxial epidermis. Data are means ± SE of six independent samples. Statistically significant differences (Tukey’s test, *p* < 0.05) between the IT and BG ecotypes are indicated by different letters.

Significant differences between IT and BG populations were found in most of the investigated anatomical traits in control (well-watered) conditions (Figure 1). IT plants from dry habitat had thicker leaves (LT) and spongy parenchyma (SP) (Figure 1A,D), while the relative area of mesophyll occupied by intercellular spaces (InS) and palisade parenchyma thickness (PP) (Figure 1B,C), as well as the epidermises (adaxial, AdE, and abaxial, AbE) (Figure 1E,F) were similar in the two populations under control conditions. 

Drought stress caused reduction in LT (Figure 1A) and PP (Figure 1C) of both populations, and the reduction was stronger in BG than in IT plants. SP thickness did not change in BG plants, while it was significantly reduced in the IT population down to a level more similar to that of BG plants (Figure 1D). AdE became thinner in BG, but did not change in IT leaves (Figure 1E). No significant changes in AbE were observed in the two drought-stressed populations, with AbE of IT again thinner than in BG leaves (Figure 1F). Finally, InS tended to decrease in BG and to increase in IT drought-stressed plants with respect to controls, but the effect was not statistically significant (Figure 1B). 

Re-watering after the drought stress induced significant increase in LT (Figure 1A), InS (Figure 1B) and SP (Figure 1D) in IT plants, up to values even higher than in controls. In BG plants this increase was less evident with only SP becoming higher than in controls but still lower than in IT plants (Figure 1D). PT increased only in IT plants to a value similar to controls, while PT of BG plants remained as low as during drought stress (Figure 1C). AdE partially recovered from the low value observed in drought-stressed BG plants (Figure 1E) whereas AbE of IT plants became thicker than in controls and drought-stressed samples, and similar to BG plants (Figure 1F).

### 2.2. Variation of Leaf Gas-Exchange and Photosynthetic Parameters

**Figure 2:**
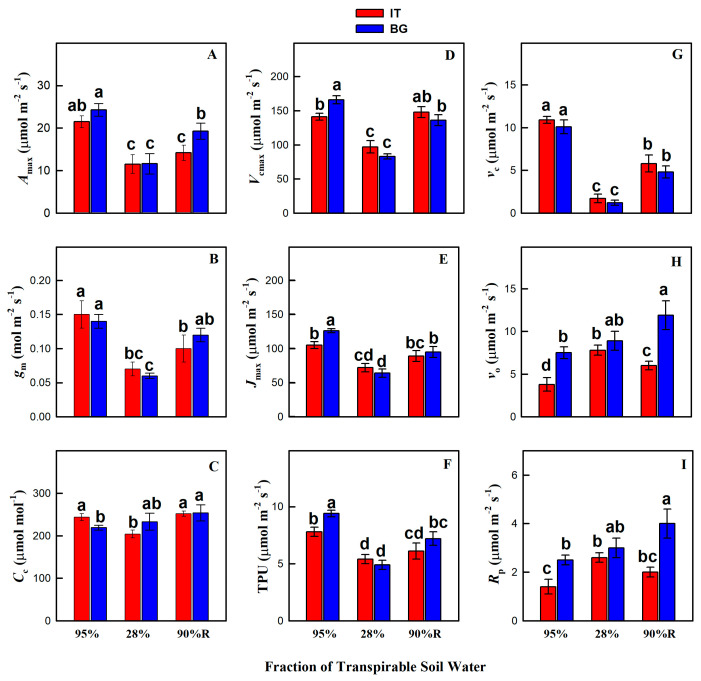
Photosynthetic parameters in Italian (IT, red bars) and Bulgarian (BG, blue bars) populations of *P. orientalis* plants under control (FTSW = 95%) and drought (FTSW = 28%) conditions and after re-watering (FTSW = 90%R). CO_2_ saturated photosynthetic rate (*A*_sat_, panel **A**), mesophyll conductance (*g*_m_, panel **B**), chloroplastic [CO_2_] at *C*_i_ = 400 ppm (*C*_c_, panel **C**), maximum carboxylation rate of Rubisco (*V*_cmax_, panel **D**), maximum rate of photosynthetic electron transport (*J*_max_, panel **E**), triose phosphate utilization (TPU, panel **F**), carboxylation rate (*v*_c_, panel **G**), oxygenation rate (*v*_o_, panel **H**) and photorespiration rate (*R*_p_, panel **I**). Data are means ± SE of eight independent samples. Statistically significant differences (Tukey’s test, *p* < 0.05) between the IT and BG ecotypes are indicated by different letters.

No significant differences were found in net photosynthetic rate calculated at saturating [CO_2_] (*A*_sat_) and mesophyll conductance (*g*_m_) (Figure 2A,B) between BG and IT plants when compared under control conditions. However, maximum carboxylation rate of Rubisco (*V*_cmax_), maximum rate of photosynthetic electron transport (*J*_max_), triose phosphate utilization (TPU), oxygenation rate (*v*_o_) and photorespiration rate (*R*_p_) were significantly higher in BG than in IT plants (Figure 2D–F,H,I), while chloroplastic [CO_2_] at *C*_i_ = 400 ppm (*C*_c_) was higher in IT than in BG control plants (Figure 2C). 

**Figure 3:**
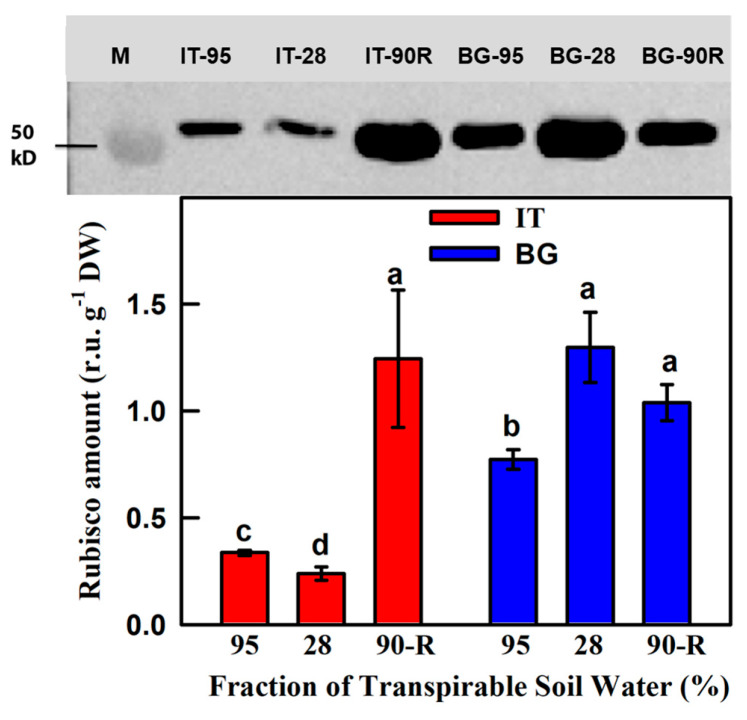
Rubisco protein content in Italian (IT, red bars) and Bulgarian (BG, blue bars) populations of *P. orientalis* plants under control (FTSW = 95%) and drought (FTSW = 28%) conditions and after re-watering (FTSW = 90%R). Rubisco protein bands were normalized to the Actin band. Data are means ± SE of six independent samples. Statistically significant differences (Tukey’s test, *p* < 0.05) between the IT and BG populations are indicated by different letters.

Most of the photosynthetic traits were negatively affected by drought. Severe stress significantly reduced *A*_sat_ and *g*_m_ (Figure 2A,B), *V*_cmax_, *J*_max_, TPU, carboxylation rate (*v*_c_) (Figure 2D–G) in both populations. *v*_o_ and *R*_p_ were stimulated by drought in both populations, especially in IT plants (Figure 3H,I). 

Re-watering generally was not accompanied by a complete recovery of the parameters (especially *v*_c_, see Figure 2G). However, *g*_m_ (Figure 2B) and *C*_c_ (Figure 2C) of BG plants and, *V*_cmax_ and *J*_max_ of IT plants (Figure 2D,E) were similar to those observed in controls. *R*_p_ (Figure 2I) and *v*_o_ (Figure 2H) remained higher or even further increased after re-watering in BG samples. 

The Rubisco protein level significantly differed between BG and IT leaves and was influenced by the treatment (Figure 3). Under well-watered conditions the amount of Rubisco protein was significantly higher in BG than in IT plants. In drought-stressed BG leaves the amount of Rubisco showed a further, significant increase, while it was significantly reduced in drought-stressed IT leaves. After re-watering, Rubisco did not revert to control values in BG leaves, and largely increased in IT leaves.

### 2.3. Variation in Leaf Antioxidants

**Figure 4:**
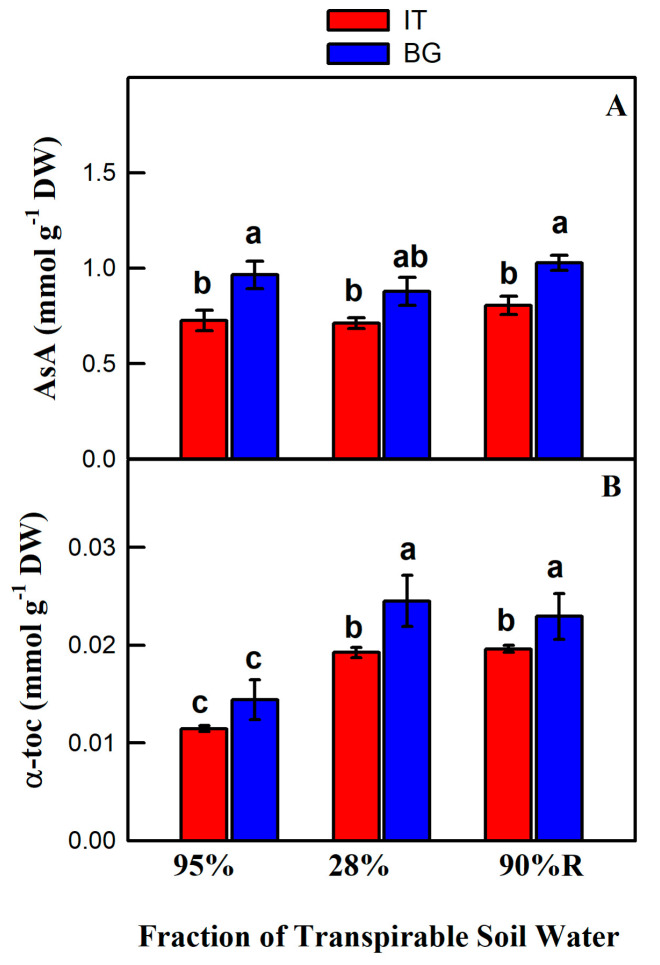
Antioxidant levels in Italian (IT, red bars) and Bulgarian (BG, blue bars) populations of *P. orientalis* plants under control (FTSW = 95%) and drought (FTSW = 28%) conditions and after re-watering (FTSW = 90%R). (**A**) Ascorbic acid; (**B**) α-tocopherol. Data are means ± SE of six independent samples. Statistically significant differences (Tukey’s test, *p* < 0.05) between the IT and BG populations are indicated by different letters.

The level of ascorbic acid and α-tocopherol were higher in BG than in IT plants under the different treatments (Figure 4). Drought did not affect the level of ascorbic acid in both populations when compared to corresponding controls (Figure 4A). However, α-tocopherol significantly increased in BG and IT plants under stressful conditions and remained higher than in control conditions after re-watering (Figure 4B).

### 2.4. Leaf Anatomical, Physiological and Biochemical Responses Analyzed by the Plasticity Index 

Leaf plastic responses of the two *P. orientalis* populations to drought and consequent re-watering were compared for different leaf anatomical, physiological and biochemical variables by means of the plasticity index. Taking into account the leaf anatomical traits, IT plants had significantly higher plasticity of SP than BG under drought, while the BG population showed significantly higher plasticity of most of other anatomical traits such as LT, PP and AdE (Table 1). Considering the overall anatomical traits analyzed, the IT population was characterized by a significantly lower plasticity to drought stress compared to the BG population (Δ_IT-BG_ = −0.040) (Table 1). The IT population also exhibited significantly lower plasticity for leaf anatomical variables compared to BG after re-watering (Δ_IT-BG_ = −0.055) (Table 1). 

The plasticity index was also calculated for physiological parameters. The plasticity of physiological responses to drought was lower in the IT than in the BG population for all variables with the only exceptions of *v*_o_ and *R*_p_ (Table 1). However, the IT population showed higher plasticity than the BG populations after re-watering (Table 1). 

Similarly, the IT population exhibited lower plastic phenotypic response than the BG population for leaf biochemical traits (Table 1). Averaging the phenotypic plasticity index for all variables, IT population showed lower value in drought conditions, and higher plasticity after re-watering compared to BG population (Table 1).

### 2.5. Correlations between Leaf Anatomy and Photosynthetic Traits

We assessed whether correlations between leaf anatomy and photosynthetic traits were affected by drought by InterCriteria Analysis (ICrA). Overall a positive correlation was observed between leaf anatomy and photosynthetic parameters in both populations when compared well-watered and drought stressed plants (Tables S1–S3) confirming previously reported observations [20,36,37]. *A*_sat_, *V*_cmax_, *J*_max_, TPU and *g*_m_ positively correlated with SP in IT plants, and in BG population positive correlation of *A*_sat_, *J*_max_ and TPU with PP was established. Thus, SP mostly contributed to the potential of photosynthetic efficiency in IT population, whereas PP reflected more the photosynthetic activity of BG plants when exposed to drought (Table S1). On the other hand, in the same experimental conditions, respiration parameters (*v*_o_ and *R*_p_) correlated negatively with LT, PP and SP only in IT samples, whereas no correlation was found in BG plants. Less correlations between parameters were found when the data collected in control and re-watered plants were considered (data not shown). Almost no relationship between structural and functional traits were found in IT population, with the exception of negative correlation between *A*_sat_ and LT and between *v*_c_ and SP. However, PP correlated positively with *A*_sat_, *J*_max_ and TPU in BG plants, but negative correlations between SP and *J*_max_, TPU and *g*_m_ were established in these samples. 

## 3. Discussion

In the present study we investigated anatomical, physiological and biochemical leaf features of two populations of *P. orientalis* originating from habitats with contrasting water availability in order to understand how drought conditions modify plant plasticity to drought and whether phenotypic differences may reinforce plant ability to resist and to be resilient to drought stress. Plant plasticity has been recognized as an important aspect of how organisms develop, function and evolve in their environment [7]. The development of adaptive traits driven by the local climate has been reported in *Pinus sylvestris* [38], *Quercus ilex*, *Q. coccifera* [39], *Quercus robur* and *Fagus silvativa* [40], *Fagus sylvatica* [41], *Quercus ilex* [42], *Corymbia calophylla* [43] and different *Picea* species [44,45]. The results of our study indicate that *P. orientalis* seedlings grown under controlled conditions from seeds of different origin are characterized by several potentially adaptive features (discussed in details bellow), as an expression of the climate of the original habitats. Our hypothesis was that populations adapted to drier environment (e.g., IT) have lost plasticity compared to populations that are not fragmented and thrive in a more humid environment (e.g., BG). It also could be expected that the climate conditions of IT decrease plasticity allowing specialization by different traits. In support of our hypothesis, we found that IT plants showed overall reduced plasticity when exposed to drought and lower anatomical plasticity after re-watering compared to BG plants (Table 1). Plant adaptation to variation in the environments often depends on genetic variability. We speculate that the reduced plasticity of IT population could be due to high genetic differentiation and low gene flow [46]. Indeed, comprehensive genetic analysis of *P. orientalis* populations from its central range (Bulgaria–Kresna, Turkey and Crete) and from isolated, marginal populations representing the westernmost edge of the species distribution in Southern Italy and Sicily showed that the central populations have higher genetic variability than western marginal once [46]. The authors concluded that the low level of genetic diversity can result from fragmentation experienced by *P. orientalis* in its westernmost distribution and may be associated with decreased adaptation potential to changing environments. 

We now analyze possible significance in terms of mechanisms of adaptation to the environment of the observed differences between the two populations, in the different experimental conditions. 

### 3.1. Leaf Anatomical Adjustments 

Differences in leaf structural characteristics of both *P. orientalis* populations, already under control conditions were found (Figure 1). The higher LT in IT plants could enable them to maintain high the relative leaf water content in the dry environment [47,48,49]. Higher LT in IT plants may also facilitate the leaf hydraulic conductance and water storage capacity [50]. IT plants also showed much more SP tissue compared to BG plants in control conditions. It was suggested that SP cells are better suited to conduct water than PP cells [50], which would give our finding an adaptive significance, facilitating water movement across the leaf in the dry environment of the IT population. On the other hand, a higher PP/SP ratio, such as that observed in BG plants, may explain higher photosynthesis of these plants in control conditions and after recovering from drought [15,51,52]. Beside facilitating water movement, higher SP may also allow better diffusion of CO_2_ to the palisade cells where CO_2_ is needed for photosynthesis [53]. In mesophytes, CO_2_ conductance through SP cells accounts for about 50% of the total *g*_m_ and considerably contributes to set leaf photosynthesis [54]. We have observed a slightly (not significantly) higher *g*_m_ in IT than in BG control plants, which however yielded significantly higher chloroplast CO_2_ concentration in IT leaves. No statistically significant differences were observed between the two populations in terms of epidermis thickness under well-watered conditions (Figure 1E,F).

Both populations responded to drought with significant reduction of LT (Figure 1A). IT plants showed similar shrinkage throughout the mesophyll, suggesting that transpirable water evaporates throughout the leaf. However, BG samples showed stronger shrinkage and potentially higher evaporation in PP [55]. The relative effect of the shrinkage of the given leaf tissues on leaf water potential is still elusive [56]. Overall changes in LT were more pronounced in BG plants compared to IT, indicating that leaf shrinkage in thickness was greater in the population from humid environment. Previously, it was shown that species native to dry habitats are more resistant to shrinkage than those originating from moist habitats, and this was due to more negative osmotic pressure and higher modulus of elasticity [57]. The authors concluded that resistance to shrinkage is an important trait contributing to drought tolerance. 

Interestingly, the leaves of IT plants became thicker than controls after re-watering. This is expected to occur in populations from drier environment that are more resistant to drought. Significantly thicker palisade of drought resistant tropical rainforest trees when exposed to soil moisture deficit did not result in higher photosynthetic capacity [37]. The SP thickness also increased significantly in both populations after re-watering, which could result in easier CO_2_ diffusion to the sites of CO_2_ fixation compared to controls [36]. While PP thickness reached control values in IT population after re-watering, it remained significantly lower in BG plants and also lower than in IT plants. Thicker PP and SP in IT population could indicate a reduced leaf area density, which in turn could allow for an improved distribution of chloroplasts and increased CO_2_ dissolution [20]. The changes in anatomical traits observed in our study may therefore represent a structural adjustment allowing adaptation of photosynthesis in response to changing environment [54]. 

### 3.2. Leaf Physiological Response 

We had previously shown that photosynthesis under ambient conditions is similar in the two genotypes and is equally affected by drought [31]. We have now further investigated possible limitations to the photosynthetic apparatus. In vivo measurements of *A/C*_i_ response curves showed that BG plants had significantly higher *V*_cmax_, *J*_max_ and TPU compared to IT plants (Figure 2D–F), indicating higher Rubisco activity (also suggested by higher Rubisco amount of BG plants, Figure 3), higher RuBP regeneration capacity and faster starch and sucrose synthesis in these control plants [58,59]. Noticeably, BG population had significantly higher *R*_p_ compared to IT plants even under well-watered conditions (Figure 2I). The higher rate of *R*_p_ in BG compared to IT plants under stress could be linked to the higher level of Rubisco protein found in these plants. High Rubisco expression in BG stressed plants may allow higher *R*_p_ as a safety mechanism against photoinhibition. Possibly higher isoprene emission of IT plants [31] might be the alternative energy sink and mechanism of energy dissipation operating when *R*_p_ is less active [60,61,62]. 

Numerous studies demonstrated that drought may induce metabolic limitations to photosynthesis [27,63,64,65,66,67,68,69]. We show a larger drop of *V*_cmax_*, J*_max_*,* TPU and *v*_c_ in BG plants, and infer that metabolic limitations to photosynthesis were stronger in BG than in IT drought-stressed plants. Drought-induced metabolic impairment of photosynthesis may also be related to protein turnover increasing protein synthesis, aggregation, denaturation or degradation. In our study, leaf Rubisco content was significantly reduced in IT plants exposed to drought (Figure 3), which might have contributed to limit *A*_sat_ (Figure 2A). By striking contrast, *A*_sat_ reduction in drought-stressed BG plants was associated with a surprising increment of Rubisco content (Figure 3). The regulation of Rubisco activity and quantity under drought stress is indeed complex and not yet well understood [70]. A strong reduction in leaf Rubisco content was detected in drought-stressed sunflower, common bean and common grape [71,72], but not in droughted barley, faba bean, *Rhamnus ludovici-salvatoris* and *Nicotiana sylvestris* [72,73], and Pääkkönen, et al. (1998) reported an increase in Rubisco content under drought in birch [74]. Clearly, in our case the observed increase of Rubisco did not influence photosynthetic performances that were otherwise limited in drought-stressed BG plants.

Under drought, the photosynthetic electron transport is often reallocated from photosynthesis to photorespiration [75], especially when *C*_c_ becomes low [76]. Indeed, drought stress stimulated the photorespiration rate by twofold in leaves of *Quercus ilex* [77] and by 25% in *Jatropha curcas* [78]. Consistent with our assessment of *C*_c_ reduction, a substantial increase of *R*_p_ and *v*_o_ (Figure 2D,H,I) was observed in drought-stressed IT plants, and to a much less extent in BG plants where *R*_p_ and *v*_o_ were high even under well-watered conditions. Stimulation of photorespiratory metabolism might be useful to protect photosynthesis especially in BG plants [79,80]. The beneficial role of *R*_p_ under abiotic stress stimuli is further strengthened by cyclic electron flow around PSI [80]. Indeed, we already reported enhanced capacity of cyclic electron flow indicated by higher intensity of thermoluminescence afterglow band in BG than in IT leaves [31]. 

After re-watering *A*_sat_ did not recover in neither plane populations. The only photosynthetic parameters reached control values in IT plants were *V*_cmax_ and *J*_max_, while TPU and *v*_o_ remained significantly inhibited. Moreover, *R*_p_ of re-watered IT plants was similar to pre-stress conditions, while if further increased in BG plants. As already speculated when comparing photosynthetic characteristics of the two populations under well-watered conditions, *R*_p_ could be one of the mechanisms in BG population to cope with unfavorable environmental conditions, and the same action could be fulfilled by the higher isoprene emission in the IT population [31]. Photorespiration can also be a source of H_2_O_2_ that may affect the redox status of plant cells [81]. *v*_o_ also remained higher in re-watered IT and BG samples. This is consistent with the incomplete recovery from drought, revealing a more permanent damage to the photosynthetic apparatus [27].

### 3.3. Leaf Biochemical Response 

H_2_O_2_ is considered a dangerous ROS. However, photorespiratory H_2_O_2_ can also act as a signal to stimulate cyclic electron flow [82]. In the presence of ROS, plants employ enzymatic and non-enzymatic mechanisms of antioxidant protection. Ascorbic acid is a main water-soluble antioxidant scavenging H_2_O_2_ [83]. α-Tocopherol, a major compound of leaf chloroplast, also deactivates photosynthesis-derived ROS (mainly ^1^O_2_ and OH^•^) and prevents lipid peroxidation by scavenging lipid peroxyl radicals in thylakoid membranes [84]. The levels of these two antioxidants were consistently higher in BG than in IT plants, indicating more active photoprotection in the BG population (Figure 4). Ascorbic acid and α-tocopherol contents also remained higher in drought-stressed and recovering BG plants compare to IT samples. The authors of [31] noted that IT plants were characterized by higher emission of isoprene compared to BG plants. As surmised above, isoprene seems to have an important antioxidant action [85] protecting photosynthetic membranes from stresses [62,86]. Thus, the two populations might have elaborated different strategies of photoprotection, with isoprene being a preferred photoprotective metabolite only in areas characterized by longer and drier summer conditions. 

## 4. Materials and Methods

### 4.1. Plant Material, Growth Conditions and Drought Treatment

*Platanus orientalis* seeds collected from native populations in Bulgaria (Kresna, 41.440800′ N, 23.082929′ E, representative of the core distribution [46]), and Italy (Francavilla di Sicilia, 37.541976′ N, 15.082318′ E, representative of the westernmost edge of distribution, Barstow and Rivers 2017) were used [31]. The locations in Bulgaria and Italy are characterized with different summer precipitations (~43 and ~14 mm, respectively, https://en.climate-data.org/location/194719/). 

Seeds germination, plant growth and drought treatment were as detailed in [31]. In brief, 14 plants of each population (28 plants in total) were grown in a climatic chamber under controlled conditions (day/night temperature 25/20 °C, light intensity 350 µmol·m^−2^·s^−1^, 14 h photoperiod, 400 µmol·mol^−1^ ambient CO_2_ concentration, and 65%–70% relative humidity) for four months. During the experiments plants were regularly watered to keep the pots to full water capacity, and were fertilized every two weeks with full-strength Hoagland solution to supply mineral nutrients at free access rates. 

Drought stress was initiated by stopping watering. The pot water content was daily controlled by calculating the fraction of transpirable soil water (FTSW, %) [87]. Measurements were performed with 4-month-old plants at three stages of the experiment: (1) under optimal water conditions before the onset of drought stress (FTSW = 95%), (2) at severe drought conditions (FTSW = 28% reached after 6–7 days of drought stress), and (3) after re-watering (recovery, FTSW = 90%R; the recovery phase was 7 days). The fourth and fifth fully expanded leaves from the top were used for all analyses. In order to assess possible age effect, four plants of each population were kept under well-watered conditions during the 14-day experimental period. No changes due to aging were observed in these plants, and therefore the data are not presented. 

### 4.2. Morpho-Anatomical Measurements

SLA was calculated as a ratio of dry mass to leaf area. Dry weight was determined from oven-dried certain area of leaf discs after 48 h at 80 °C. 

For anatomical studies, leaf pieces taken from the middle part of fully expanded 4th leaves of well-watered, drought-stressed and re-watered plants were fixed in 3% (*m*/*v*) glutaraldehyde buffered with 0.1 M sodium phosphate to pH 7.4. Hand-made transversal sections (at least 30 per species) were mounted on slides in glycerol and examined with a light microscope. Images were collected through a digital camera (Nikon Eclipse 50i, Tokyo, Japan) and analyzed using ImageJ (National Institutes of Health, Bethesda, MD, USA). Leaf anatomy was characterized by measuring the thickness of leaf (LT), spongy parenchyma (SP), palisade parenchyma (PP) and of both adaxial (AdE) and abaxial (AbE) epidermis. The relative area of mesophyll occupied by intercellular spaces (InS) was calculated as *S*_i_/*S*_m_, where S_i_ is the surface area occupied by intercellular spaces and *S*_m_ is the mesophyll surface area analyzed.

### 4.3. Photosynthetic Measurements

Leaf photosynthetic gas exchange was evaluated with a portable gas exchange system (LCpro+, ADC BioScientific, UK). Photosynthesis responses to intercellular CO_2_ concentrations (*A*/*C*_i_ curves) were analyzed [88]. Leaf in the cuvette was exposed to a range of [CO_2_] growing from 50 to 1800 μmol mol^−1^. All gas exchange parameters were recorded after reaching the steady-state photosynthesis for each [CO_2_], usually 5–10 min after the change of external [CO_2_] (*C*_a_). Measurements were performed at 25 ± 1 °C leaf temperature, 800 µmol m^−2^ s^−1^ photosynthetic photon flux density (PPFD) at the leaf level and 45–50% relative humidity in the leaf cuvette. The CO_2_ saturated photosynthesis (*A*_sat_, μmol·m^−2^·s^−1^), the maximum carboxylation rate allowed by ribulose1,5 bisphosphate carboxylase/oxygenase (Rubisco) (*V*_cmax_, μmol·m^−2^·s^−1^), the maximum rate of photosynthetic electron transport based on NADPH requirement for ribulose 1,5 bisphosphate (RuBP) regeneration (*J*_max_, μmol·m^−2^·s^−1^) and triose phosphate utilization (TPU, μmol·m^−2^·s^−1^) were calculated using the method of [89] from *A*/*C*_i_ data. Mesophyll conductance (*g*_m_) (i.e., the conductance to CO_2_ inside leaves, between intercellular spaces and the chloroplasts) was determined using the variable J described by [90,91]. Oxygen concentration was lowered to 2% when testing leaf gas exchange under non-photorespiratory conditions as described in [17]. The [CO_2_] at the chloroplast sites (*C_c_*) was calculated at *C*_i_ = 400 ppm using g_m_ as shown by [90]. Photorespiration (*R*_p_), carboxylation (*v*_c_) and oxygenation (*v*_o_) rates were calculated according to [76].

### 4.4. Water-Soluble and Fat-Soluble Antioxidants

For the evaluation of total antioxidant capacity, leaf samples were cut, sonicated and resuspended in a volume of methanol/water/formic acid (80:20:0.1; *v*/*v*/*v*). The suspensions were homogenized, shaken for 2 h at room temperature in dark condition. After centrifugation at 3500 rpm for 15 min, the pellets were resuspended and homogenized in another volume of solvent and centrifuged once more. The supernatants were combined with the first extract and labelled as the water-soluble extract. The pellets were used to extract fat-soluble antioxidants: They were resuspended in a volume of acetone, shaken at room temperature and centrifuged at 3500 rpm for 5 min. The first supernatants were transferred to new tubes, while the pellets were re-extracted following the same procedure. The second supernatants were combined with the first extracts and labelled as fat-soluble extracts. The water-soluble and fat-soluble extracts were kept at 4 °C until immediate use in the spectrophotometric determination of antioxidant capacity. Total antioxidant capacity was measured as described in [92] modified by [93]. Aliquots of 0.1 mL of water-soluble or fat-soluble extracts were combined with 1 mL of reagent solution (0.6 M sulfuric acid, 28 mM sodium phosphate, and 4 mM ammonium molybdate). Water-soluble extracts were incubated at 95 °C for 90 min, while fat-soluble extracts were incubated at 37 °C for 90 min in a water bath with constant shaking. The samples were cooled to room temperature, and the absorbance was measured at 695 nm. A blank solution, containing 1 mL of reagent solution and the same volume of solvent used for the samples, was incubated together with samples and used as a reference. Stock solutions of ascorbic acid and α-tocopherol were used for the calibration curves. Water-soluble and fat-soluble antioxidant capacity was expressed as equivalents of ascorbic acid (mmol·g^−1^ DW) and as equivalents of α-tocopherol (mmol·g^−1^ DW), respectively.

### 4.5. Leaf Protein Extraction and Rubisco Determination

For Rubisco (RuBP) quantification, leaf proteins were extracted following the procedure of [94] as described in [95]. A 12% dodecyl sulfate-polyacrylamide gel electrophoresis (SDS-PAGE) was performed using 5 μg of protein samples, Dual Color Protein Standard (Bio-Rad Laboratories, Milan, Italy) as a marker and Laemmli loading buffer to follow protein separation.

Western blot analysis was performed using a blocking solution (100 mM Tris-HCl pH 8.0, 150 mM NaCl, 0,1% Tween 20, 5% BSA) and primary antibodies (Agrisera, Vännäs, Sweden) to reveal Rubisco (anti-RbcL, rabbit polyclonal serum), and Actin (anti-ACT, rabbit polyclonal serum) as a loading control. A kit for chemiluminescence (Westar Supernova, Cyanagen, Bologna, Italy) was used for immunorevelation in a ChemiDoc System (Bio-Rad). Densitometry analysis was performed using ImageJ software (Rasband, W.S., U.S. NIH, Bethesda, Maryland, USA, 1997–2012) and results were expressed in arbitrary units and referred to dry leaf weight.

### 4.6. Plasticity Index

The phenotypic plasticity index (PI) was calculated for each measured variable as the difference between maximum and minimum values divided by the maximum value [39].

PI = [(maximum value − minimum value)/maximum value](1)

To quantify the degree of phenotypic plasticity in response to stress (PI_stress_) the values of the traits measured in control plants and drought-stressed plants were considered. To calculate plasticity index in recovered plants (PI_recovery_) the trait values measured in control plants and in plants after re-watering were taken into account. PI ranges from 0 (no plasticity) to 1 indicating high plasticity.

### 4.7. Statistical Analyses

Averages and standard errors (*n* = 5–8 biological replicates) of all parameters are reported. Analyses of variance were performed to test the significant differences between treatments (control, drought and recovery). These differences were separated by a Tukey’s test, and those significantly different at the 5% level (*p* < 0.05) are shown by different letters. A Student’s *t*-test was used to determine the significant differences in PI only within groups (PI_stress_, IT and BG populations exposed to drought; and PI_recovery_, IT and BG populations after re-watering). Asterisks indicate significant differences as follow * *p* < 0.1, ** *p* < 0.05 and *** *p* < 0.01.

InterCriteria Analysis (ICrA) was used to evaluate the relationships between measured parameters. It is a novel approach for decision support, based on Indexed Matrices [96] and Intuitionistic Fuzzy Sets [97]. ICrA has been successfully applied in various fields as biology/medicine [98,99], algorithms performance [100,101,102], fuel industry [103] and economics [104]. ICrA advantage over other correlation analyses (e.g., Pearson’s correlation coefficient, which refers to linear relationship) is that it also considers nonlinear dependencies between parameters. Using ICrA, arrays of data obtained by the measurement of specific objects (5–8 biological replicates) against defined criteria (measured parameters) are processed to define dependence (correlation) between the criteria themselves. The cross-platform software ICrAData was used [105]. The ICrA calculates the pairwise relationship between each pair of criteria in the form of intuitionistic fuzzy pairs—degree of “agreement” $\mu_{C_{k},C_{l}}$ (degree of relationship between parameter $C_{k}$ and parameter $C_{l}$) and degree of “disagreement” $\nu_{C_{k},C_{l}}$. (degree of no relationship between $C_{k}$ and $C_{l}$) [106]. The complement $1-\mu_{C_{k},C_{l}}-\nu_{C_{k},C_{l}}=\pi_{C_{k},C_{l}}$ represents the degree of “uncertainty” (degree of unreliability of the results).

## 5. Conclusions

Climate changes are likely to exert pressure on the adaptive capacity of the next generations of forest trees. However, these changes are so fast that current tree generations also need to resist and/or acclimate quickly [107]. This is particularly important for species with fragmented distribution like *P. orientalis*, as isolated populations growing in enclaves are characterized by lower genetic variability [46]. The important role of environmental factors in seed and progeny fitness in fragmented environments was already highlighted by [108,109], and it could be expected that plant species will develop adaptive features to help them cope with adverse environmental conditions. This study revealed that the Italian endangered population which is adapted to drier environment showed overall lower plasticity when exposed to drought stress, confirming our hypothesis that plasticity is lost in populations fragmented and isolated in harsher environments. The lower plasticity of IT plants not only under drought conditions but also after re-watering is likely associated with the development of leaf traits (e.g., thicker leaves) that allow these plants to survive under long dry periods (summers), whereas, the BG population relies on physiological and biochemical plasticity in order to cope with and be resilient to shorter drought events. Our results suggest that phenotypic plasticity can provide insights on the mechanisms of adaptation to specific environmental conditions and could yield useful information about how taxa with fragmented distribution will respond to the forecasted climate changes.

## Figures and Tables

**Table 1:** Phenotypic plasticity index of Italian (IT) and Bulgarian (BG) *P. orientalis* populations in response to drought (PI stress) and after re-watering (PI recovery) for the variables of Figure 1, Figure 2, Figure 3 and Figure 4.

Parameter	PI Stress				PI Recovery		
	IT	BG	Δ_IT−BG_		IT	BG	Δ_IT−BG_
***Leaf Anatomical Traits***							
**LT**	0.112 ± 0.008	0.170 ± 0.018 **	−0.058		0.083 ± 0.018	0.035 ± 0.007 **	0.048
**PP**	0.130 ± 0.035	0.225 ± 0.038 *	−0.096		0.056 ± 0.015	0.183 ± 0.036 ***	−0.127
**SP**	0.189 ± 0.025	0.074 ± 0.012 ***	0.115		0.138 ± 0.031	0.243 ± 0.052 *	−0.104
**AdE**	0.100 ± 0.019	0.287 ± 0.057 ***	−0.187		0.055 ± 0.025	0.178 ± 0.032 ***	−0.123
**AbE**	0.157 ± 0.027	0.165 ± 0.050	−0.008		0.145 ± 0.043	0.160 ± 0.014	−0.014
**InS**	0.147 ± 0.046	0.152 ± 0.024	−0.005		0.159 ± 0.011	0.171 ± 0.057	−0.012
**Sub-Total**	**0.139 ± 0.012**	0.179 ± 0.010 **	**−0.040**		**0.106 ± 0.009**	0.162 ± 0.021 **	**−0.055**
***Leaf Physiological Traits***							
***A*_sat_**	0.450 ± 0.112	0.518 ± 0.095	−0.068		0.338 ± 0.077	0.209 ± 0.060	0.128
***V*_cmax_**	0.321 ± 0.042	0.496 ± 0.034 ***	−0.175		0.126 ± 0.025	0.200 ± 0.048	−0.074
***J*_cmax_**	0.314 ± 0.061	0.485 ± 0.055 **	−0.172		0.191 ± 0.043	0.246 ± 0.061	−0.055
**TPU**	0.315 ± 0.058	0.470 ± 0.048 **	−0.155		0.264 ± 0.046	0.239 ± 0.063	0.025
***v*_c_**	0.842 ± 0.045	0.879 ± 0.034	−0.037		0.521 ± 0.059	0.516 ± 0.059	0.004
***v*_o_**	0.501 ± 0.093	0.255 ± 0.080 *	0.246		0.399 ± 0.107	0.364 ± 0.059	0.035
***R*_p_**	0.441 ± 0.084	0.210 ± 0.050 **	0.231		0.304 ± 0.109	0.364 ± 0.059	−0.060
***g*_m_**	0.562 ± 0.068	0.550 ± 0.071	0.012		0.405 ± 0.068	0.193 ± 0.051 **	0.212
**Rubisco**	0.286 ± 0.030	0.405 ± 0.029 **	−0.119		0.724 ± 0.024	0.253 ± 0.056 ***	0.470
**Sub-Total**	**0.459 ± 0.043**	**0.480 ± 0.017**	**−0.021**		**0.336 ± 0.036**	**0.280 ± 0.034**	**0.056**
***Leaf Biochemical Traits***							
**AsA**	0.146 ± 0.007	0.226 ± 0.018	−0.081		0.229 ± 0.021	0.200 ± 0.021	0.029
**α-toc**	0.402 ± 0.034	0.379 ± 0.041	0.023		0.415 ± 0.016	0.338 ± 0.064	0.078
**Sub-Total**	**0.273 ± 0.022**	**0.331 ± 0.050**	**−0.058**		**0.322 ± 0.024**	**0.260 ± 0.052**	**0.062**
**TOTAL**	**0.338 ± 0.029**	**0.376 ± 0.018**	**−0.039**		**0.264 ± 0.017**	**0.243 ± 0.027**	**0.021**

Asterisks indicate different levels of significance within PI stress and PI recovery groups (* *p <* 0.1, ** *p <* 0.05 and *** *p <* 0.01). Lower or higher plasticity of IT population compared to BG plants is presented as negative or positive Δ_IT−BG_ values, respectively.

## Supplementary Materials

The following are available online at https://www.mdpi.com/1422-0067/21/11/3912/s1. Tables S1–S3 provide values for the degree of agreement $\mu_{C_{k},C_{l}}$ disagreement $\nu_{C_{k},C_{l}}$ and the degree of uncertainty $\pi_{C_{k},C_{l}}$, respectively, between the leaf anatomical and physiological parameters determined in two populations of *Platanus orientalis* (IT and BG) under control (FTSW = 95%) and drought (FTSW = 28%) conditions.
