# Supplementary material for: Functional and Structural Leaf Plasticity Determine Photosynthetic Performances during Drought Stress and Recovery in Two Platanus orientalis Populations from Contrasting Habitats
Source: Int J Mol Sci. 2020 May 30;21(11):3912. doi: 10.3390/ijms21113912 (PMC7312932; doi:10.3390/ijms21113912)
Supplement: Supplementary file 1 [file ijms-21-03912-s001.pdf]

Table S1. Values for the degree of agreement μ_{C_k,C_l} between the leaf anatomical and physiological parameters determined in Italian (IT) and Bulgarian (BG) populations of *P. orientalis* plants under control (FTSW = 95%) and drought (FTSW = 28%) conditions. LT – leaf thickness, PP – palisade parenchyma, SP – spongy parenchyma, AdE – adaxial epidermis, AbE – abaxial epidermis, InS – intercellular space, A_{sat} – CO_2 saturated photosynthetic rate, V_{cmax} – maximum carboxylation rate of Rubisco, J_{max} – maximum rate of photosynthetic electron transport, TPU – triose phosphate utilization, v_c – carboxylation rate, v_o – oxygenation rate, R_p – photorespiration, g_m - mesophyll conductance. The IT population data are shown in upper triangle of the table, while those for BG population – in the lower triangle. The degrees of agreement showing positive correlations are presented in blue color, those showing the negative correlations – in red color.

μ_{C_k,C_l}	LT	PP	SP	AdE	AbE	InS	A_{sat}	V_{cmax}	J_{max}	TPU	v_c	v_o	R_p	g_m
**LT**		**0.82**	0.7	0.61	0.52	0.35	0.68	**0.85**	**0.76**	0.71	**0.86**	**0.20**	**0.17**	0.71
**PP**	0.71		0.7	0.52	0.48	0.44	0.65	0.7	0.67	0.65	0.74	**0.14**	**0.17**	0.71
**SP**	0.59	0.55		0.47	0.45	0.35	**0.80**	**0.82**	**0.79**	**0.77**	0.62	**0.20**	**0.2**	**0.80**
**AdE**	0.71	0.73	0.48		0.42	0.45	0.47	0.58	0.58	0.52	0.65	0.45	0.45	0.45
**AbE**	0.55	0.38	0.62	0.44		0.59	0.5	0.45	0.42	0.44	0.47	0.56	0.5	0.55
**InS**	0.55	0.65	0.5	0.44	0.52		**0.15**	0.29	**0.17**	**0.21**	0.27	0.61	0.58	0.33
A_{sat}	**0.79**	**0.89**	0.56	0.65	0.42	0.67		**0.80**	**0.86**	**0.83**	0.7	0.3	0.33	**0.77**
V_{cmax}	**0.76**	0.71	0.5	**0.80**	0.39	0.45	**0.76**		**0.85**	**0.83**	**0.80**	**0.23**	**0.23**	**0.80**
J_{max}	**0.77**	**0.85**	0.58	**0.76**	0.35	0.56	**0.83**	0.74		**0.92**	**0.77**	0.32	0.35	0.74
**TPU**	**0.77**	**0.80**	0.55	**0.76**	0.35	0.55	**0.82**	**0.76**	**0.95**		0.74	0.32	0.35	**0.76**
v_c	**0.82**	0.74	0.53	**0.77**	0.48	0.52	**0.76**	0.73	**0.83**	**0.86**		0.27	**0.24**	0.71
v_o	0.41	0.55	0.58	0.42	0.5	0.59	0.53	0.47	0.42	0.38	0.32		**0.94**	0.32
R_p	0.41	0.55	0.58	0.42	0.5	0.59	0.53	0.47	0.42	0.38	0.32	**1.00**		0.26
g_m	0.73	0.7	0.41	**0.76**	0.35	0.5	0.7	0.74	**0.76**	**0.77**	**0.76**	0.32	0.32	

Table S2. Values for the degree of disagreement ν_{C_k,C_l} between the leaf anatomical and physiological parameters determined in Italian (IT) and Bulgarian (BG) populations of *P. orientalis* plants under control (FTSW = 95%) and drought (FTSW = 28%) conditions. LT – leaf thickness, PP – palisade parenchyma, SP – spongy parenchyma, AdE – adaxial epidermis, AbE – abaxial epidermis, InS – intercellular space, A_{sat} – CO_2 saturated photosynthetic rate, V_{cmax} – maximum carboxylation rate of Rubisco, J_{max} – maximum rate of photosynthetic electron transport, TPU – triose phosphate utilization, v_c – carboxylation rate, v_o – oxygenation rate, R_p – photorespiration, g_m - mesophyll conductance. The IT population data are shown in upper triangle of the table, while those for BG population – in the lower triangle. The degrees of disagreement are presented in the same colours as the corresponding values of the degree of agreement.

ν_{C_k,C_l}	**LT**	**PP**	**SP**	**AdE**	**AbE**	**InS**	A_{sat}	V_{cmax}	J_{max}	**TPU**	v_c	v_o	R_p	g_m
**LT**		**0.18**	0.3	0.38	0.48	0.65	0.32	**0.15**	**0.24**	0.27	**0.14**	**0.80**	**0.83**	0.27
**PP**	0.29		0.3	0.47	0.52	0.56	0.35	0.3	0.33	0.33	0.26	**0.86**	**0.83**	0.27
**SP**	0.41	0.45		0.52	0.55	0.65	**0.20**	**0.18**	**0.21**	**0.21**	0.38	**0.80**	**0.80**	**0.18**
**AdE**	0.29	0.27	0.52		0.56	0.53	0.52	0.41	0.41	0.45	0.33	0.53	0.53	0.52
**AbE**	0.45	0.62	0.38	0.56		0.41	0.50	0.55	0.58	0.55	0.53	0.44	0.5	0.44
**InS**	0.45	0.35	0.5	0.56	0.48		**0.85**	0.71	**0.83**	**0.77**	0.73	0.39	0.42	0.65
A_{sat}	**0.21**	**0.11**	0.44	0.35	0.58	0.33		**0.20**	**0.14**	**0.15**	0.3	0.7	0.67	**0.21**
V_{cmax}	**0.24**	0.29	0.5	**0.20**	0.61	0.55	**0.24**		**0.15**	**0.15**	**0.20**	**0.77**	**0.77**	**0.18**
J_{max}	**0.23**	**0.15**	0.42	**0.24**	0.65	0.44	**0.17**	0.26		**0.06**	**0.23**	0.68	0.65	0.24
**TPU**	**0.21**	**0.18**	0.44	**0.23**	0.64	0.44	**0.17**	**0.23**	**0.03**		0.24	0.67	0.64	**0.21**
v_c	**0.18**	0.26	0.47	**0.23**	0.52	0.48	**0.24**	0.27	**0.17**	**0.12**		0.73	**0.76**	0.27
v_o	0.59	0.45	0.42	0.58	0.5	0.41	0.47	0.53	0.58	0.61	0.68		**0.06**	0.67
R_p	0.59	0.45	0.42	0.58	0.5	0.41	0.47	0.53	0.58	0.61	0.68	**0**		0.73
g_m	0.18	0.21	0.5	**0.15**	0.56	0.41	0.21	0.17	**0.15**	**0.12**	**0.15**	0.59	0.59	

Table S3. Values for the degree of uncertainty π_{C_k,C_l} between the leaf anatomical and physiological parameters determined in Italian (IT) and Bulgarian (BG) populations of *P. orientalis* plants under control (FTSW = 95%) and drought (FTSW = 28%) conditions. LT – leaf thickness, PP – palisade parenchyma, SP – spongy parenchyma, AdE – adaxial epidermis, AbE – abaxial epidermis, InS – intercellular space, A_{sat} – CO_2 saturated photosynthetic rate, V_{cmax} – maximum carboxylation rate of Rubisco, J_{max} – maximum rate of photosynthetic electron transport, TPU – triose phosphate utilization, v_c – carboxylation rate, v_o – oxygenation rate, R_p – photorespiration, g_m - mesophyll conductance. The IT population data are shown in upper triangle of the table, while those for BG population – in the lower triangle. The degrees of uncertainty are presented in the same colours as the corresponding values of the degree of agreement.

π_{C_k,C_l}	LT	PP	SP	AdE	AbE	InS	A_{sat}	V_{cmax}	J_{max}	TPU	v_c	v_o	R_p	g_m
**LT**		**0**	0	0.01	0	0	0	**0**	**0**	0.02	**0**	**0**	**0**	0.02
**PP**	0		0	0.01	0	0	0	0	0	0.02	0	**0**	**0**	0.02
**SP**	0	0		0.01	0	0	**0**	**0**	**0**	**0.02**	0	**0**	**0**	**0.02**
**AdE**	0	0	0		0.02	0.02	0.01	0.01	0.01	0.03	0.02	0.02	0.02	0.03
**AbE**	0	0	0	0		0	0	0	0	0.01	0	0	0	0.01
**InS**	0	0	0	0	0		**0**	0	**0**	**0.02**	0	0	0	0.02
A_{sat}	**0**	**0**	0	0	0	0		**0**	**0**	**0.02**	0	0	0	**0.02**
V_{cmax}	**0**	0	0	**0**	0	0	**0**		**0**	**0.02**	**0**	**0**	**0**	**0.02**
J_{max}	**0**	**0**	0	**0**	0	0	**0**	0		**0.02**	**0**	0	0	0.02
**TPU**	**0.02**	**0.02**	0.01	**0.01**	0.01	0.01	**0.01**	**0.01**	**0.02**		0.02	0.01	0.01	**0.03**
v_c	**0**	0	0	**0**	0	0	**0**	0	**0**	**0.02**		0	**0**	0.02
v_o	0	0	0	0	0	0	0	0	0	0.01	0		**0**	0.01
R_p	0	0	0	0	0	0	0	0	0	0.01	0	**0**		0.01
g_m	0.09	0.09	0.09	**0.09**	0.09	0.09	0.09	0.09	**0.09**	**0.11**	**0.09**	0.09	0.09	
